# Protective effects of growth hormone-releasing hormone analogs in DSS-induced colitis in mice

**DOI:** 10.1038/s41598-021-81778-4

**Published:** 2021-01-28

**Authors:** Lucia Recinella, Annalisa Chiavaroli, Valentina Di Valerio, Serena Veschi, Giustino Orlando, Claudio Ferrante, Iacopo Gesmundo, Riccarda Granata, Renzhi Cai, Wei Sha, Andrew V. Schally, Rossano Lattanzio, Luigi Brunetti, Sheila Leone

**Affiliations:** 1 Department of Pharmacy, G. d’Annunzio University of Chieti-Pescara, Via dei Vestini 31, 66100 Chieti, Italy; 2 Department of Medicine and Ageing Sciences, G. d’Annunzio University of Chieti-Pescara, Chieti, Italy; 3 Division of Endocrinology, Diabetes and Metabolism, Department of Medical Sciences, University of Turin and Città Della Salute e Della Scienza Hospital, Turin, Italy; 4 Veterans Affairs Medical Center, Miami, FL 33125 USA; 5 Division of Endocrinology, Diabetes and Metabolism, and Division of Medical/Oncology, Department of Medicine, and Department of Pathology, Miller School of Medicine, University of Miami, Miami, FL 33136 USA; 6 Sylvester Comprehensive Cancer Center, Miami, FL 33136 USA; 7 Department of Medical, Oral and Biotechnological Sciences, G. d’Annunzio University of Chieti-Pescara, Chieti, Italy; 8 Center for Advanced Studies and Technology (CAST), G. d’Annunzio University of Chieti-Pescara, Chieti, Italy

**Keywords:** Molecular biology, Gastroenterology

## Abstract

Besides its metabolic and endocrine effects, growth hormone (GH)-releasing hormone (GHRH) is involved in the modulation of inflammation. Recently synthetized GHRH antagonist MIA-690 and MR-409, GHRH agonist, developed by us have shown potent pharmacological effects in various experimental paradigms. However, whether their administration modify resistance to chronic inflammatory stimuli in colon is still unknown. Ex vivo results demonstrated that MIA-690 and MR-409 inhibited production of pro-inflammatory and oxidative markers induced by lipopolysaccharide on isolated mouse colon specimens. In vivo, both MIA-690 and MR-409 have also been able to decrease the responsiveness to nociceptive stimulus, in hot plate test. Additionally, both peptides also induced a decreased sensitivity to acute and persistent inflammatory stimuli in male mice, in formalin test and dextran sodium sulfate (DSS)-induced colitis model, respectively. MIA-690 and MR-409 attenuate DSS-induced colitis with particular regard to clinical manifestations, histopathological damage and release of pro-inflammatory and oxidative markers in colon specimens. Respect to MR-409, MIA-690 showed higher efficacy in inhibiting prostaglandin (PG)E_2_, 8-iso-PGF_2α_ and serotonin (5-HT) levels, as well as tumor necrosis factor (TNF)-α, interleukin (IL)-6 and nitric oxide synthase gene expression in colon specimens of DSS-induced colitis. Furthermore, MIA-690 decreased serum insulin-like growth factor (IGF)-1 levels in mice DSS-treated, respect to MR-409. Thus, our findings highlight the protective effects of MIA-690 and MR-409 on inflammation stimuli. The higher antinflammatory and antioxidant activities observed with MIA-690 could be related to decreased serum IGF-1 levels.

## Introduction

The hypothalamic peptide growth hormone-releasing hormone (GHRH) stimulates the production and secretion of growth hormone (GH) by binding to pituitary type GHRH receptor (GHRH-R)^1^. GHRH also exerts direct extrapituitary activities^2,3^, such as cardioprotection^4,5^, regeneration of pancreatic islets^6^, wound healing^7^, as well as survival and antiapoptotic effects^8,9^. In addition, GHRH acts as an autocrine and/or paracrine growth factor in normal non-neoplastic cells and in cancers through the involvement of GHRH-R and its splice variant type 1 (SV1)^1–3,10–12^.

Several agonistic and antagonistic analogs of GHRH have been synthesized by our group and others, and evaluated for their biological activities^11,13–19^. In particular, the new generation GHRH antagonists of the Miami (MIA) series, MIA-690 and MIA-602, were shown able to inhibit growth of various human cancer cells and xenografts^2,18,20^. The most powerful antitumor analogs, MIA-690 and MIA-602 also exerted antinflammatory activities^18,19,21^. On the other hand, the MIA-series of GHRH analogs displayed increased GHRH-R binding affinity along with a weak GH inhibitory activity on pituitary somatotrophs^18^. GHRH agonists of MR series, including MR-409, after subcutaneous administration showed increased potency and greater binding activity compared with the parent hormone^2,17^. Recent studies demonstrated that MR-409 inhibits the in vivo growth of lung cancer xenografted into nude mice^17,19^.

In this study, we studied the possible antinflammatory and antioxidant properties of GHRH antagonist, MIA-690, and agonist, MR-409 both ex vivo, through incubation of colon sections with bacterial lipopolysaccaride (LPS) and in vivo, following dextran sodium sulfate (DSS) treatment.

## Results

### Inhibitory effects of MIA-690 and MR-409 on LPS-induced prostaglandin (PG)E_2_ and 8-iso-PGF_2α_ levels in colon specimens

**Figure 1:**
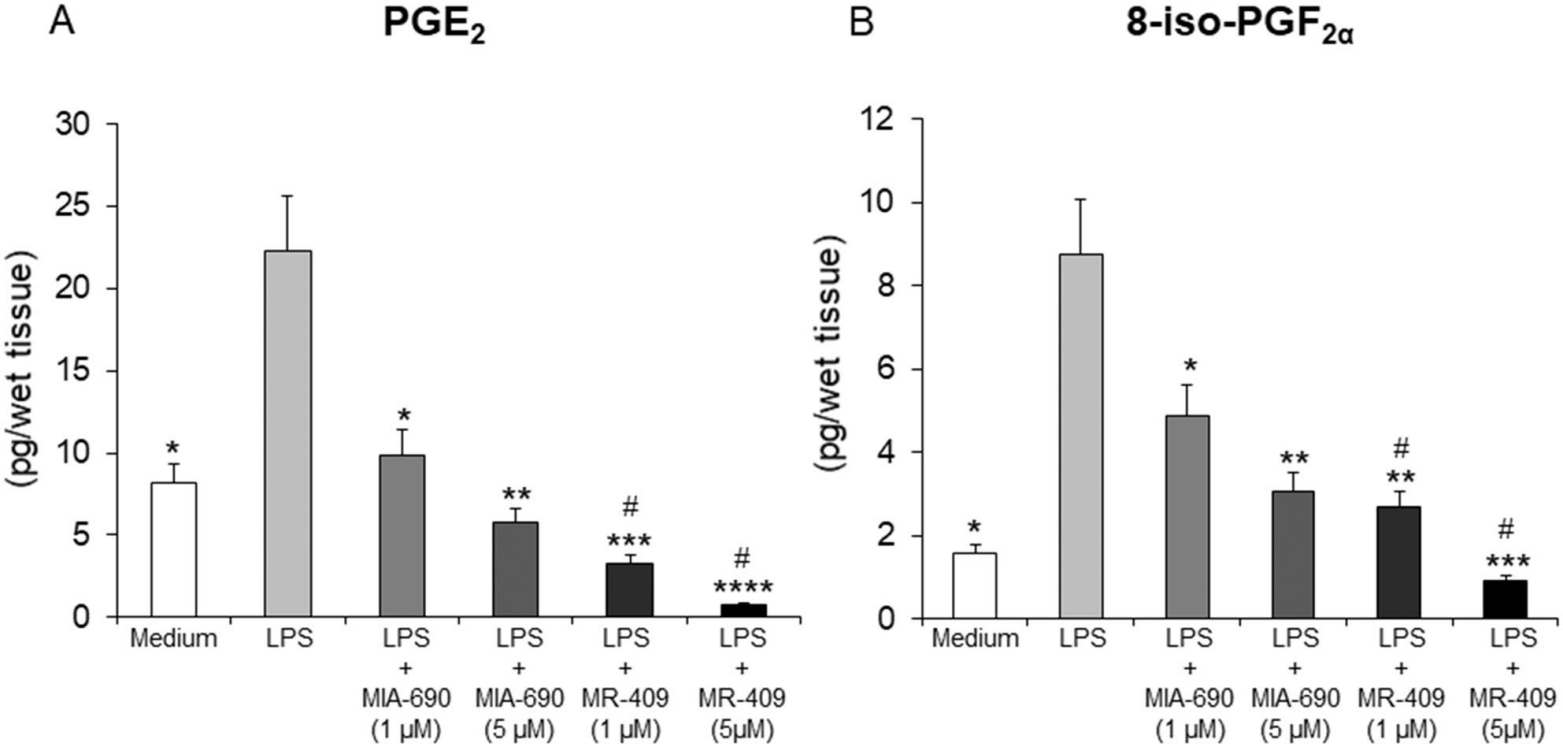
Inhibitory effects of MIA-690 (1 and 5 μM) and MR-409 (1 and 5 μM) on PGE_2_ (**A**) and 8-iso-PGF_2α_ (**B**) levels, ex vivo (n = 5 for each group of treatment). Data are expressed as means ± S.E.M. and analyzed by analysis of variance (ANOVA) followed by Bonferroni *post-hoc* test *p < 0.05, **p < 0.01, ***p < 0.005, ****p < 0.001 versus LPS; ^#^*p* < 0.05 versus treatment with MIA-690.

PGE_2_ and 8-iso-PGF_2α_ levels from tissue supernatants were measured by RIA, after treatment of colon specimens with LPS + MIA-690 (1 and 5 μM), LPS + MR-409 (1 and 5 μM), LPS or vehicle. We found significantly elevated PGE_2_ and 8-iso-PGF_2α_ levels in colon specimens, following treatment with LPS, as compared with vehicle-treated controls. The GHRH antagonist MIA-690 (1 and 5 μM) and GHRH agonist MR-409 (1 and 5 μM) were shown to decrease LPS-induced PGE_2_ and 8-iso-PGF_2α_ levels, in a dose-dependent manner [Fig. 1 panel A and B; *p* < 0.05 and *p* < 0.01 (for MIA-690); *p* < 0.005 and *p* < 0.001 (for MR-409)]. MR-409 (1 and 5 μM) was more effective than MIA-690 in inhibiting LPS-induced PGE_2_ and 8-iso-PGF_2α_ levels [Fig. 1 panel A and B; *p* < 0.05].

### Inhibitory effects of MIA-690 and MR-409 on LPS-induced lactate dehydrogenase (LDH) activity and nitrite production in colon specimens

**Figure 2:**
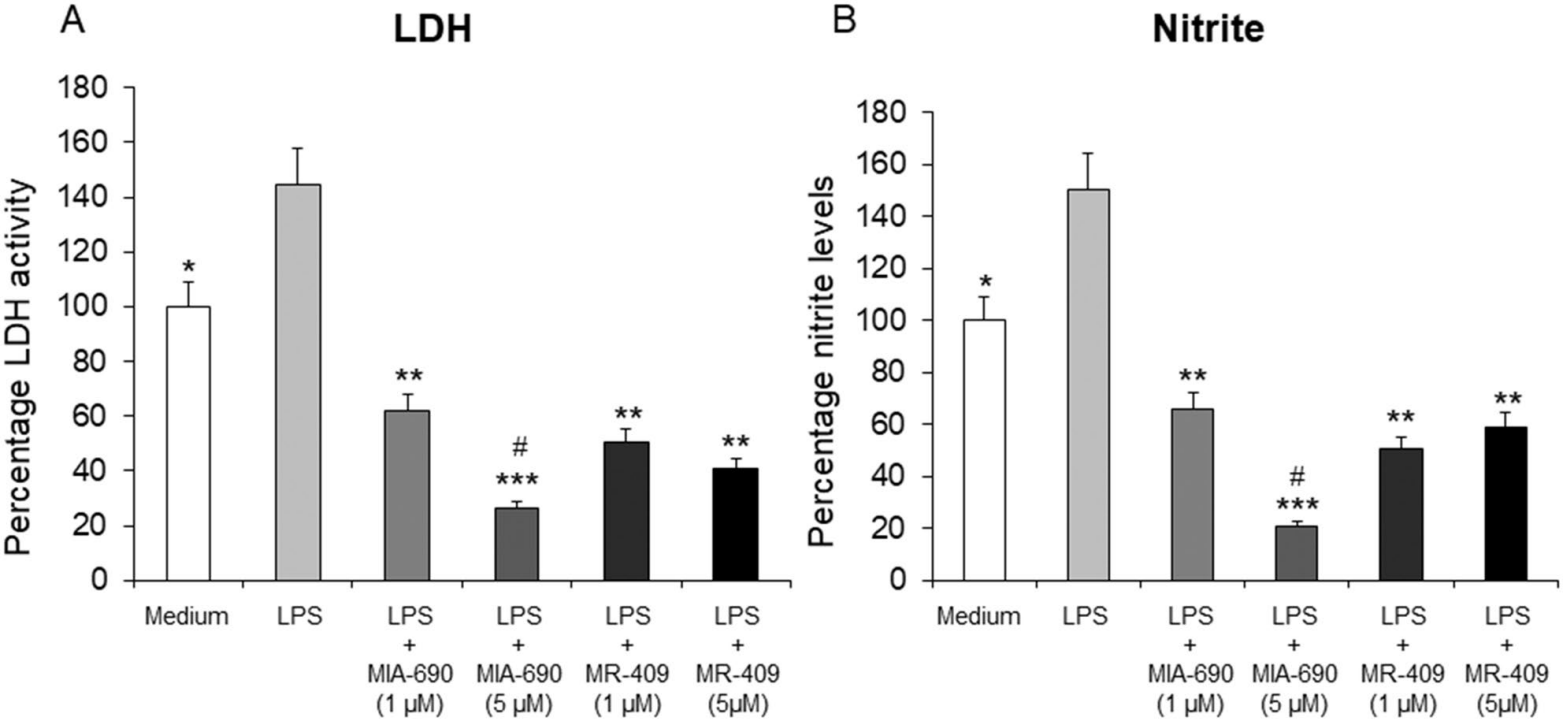
Inhibitory effects of MIA-690 (1 and 5 μM) and MR-409 (1 and 5 μM) on LDH activity (**A**) and nitrite production (**B**), ex vivo (n = 5 for each group of treatment). Data are expressed as means ± S.E.M. and analyzed by analysis of variance (ANOVA) followed by Bonferroni *post-hoc* test *p < 0.05, **p < 0.01, ***p < 0.005 versus LPS; ^#^*p* < 0.05 versus treatment with MR-409.

In order to investigate the possible effects of MIA-690 (1 and 5 μM) and MR-409 (1 and 5 μM) on oxidative stress biomarkers, we evaluated LPS-induced LDH activity and nitrite production in colon specimens following treatment with the GHRH analogs. LPS treatment significantly stimulated LDH activity and nitrite production, respect to vehicle-treated controls. MIA-690 (1 and 5 μM) reduced LDH activity and nitrite levels in a dose-dependent manner (Fig. 2 panel A and B; *p* < 0.01 and *p* < 0.005). Moreover, MR-409 (1 and 5 μM) was found able to decrease LPS-induced LDH activity and nitrite production, without showing a dose-dependent effect [Fig. 2 panel A and B; *p* < 0.01]. MIA-690 (5 μM) was more effective in inhibiting LDH and nitrite production after LPS treatment respect to MR-409 [Fig. 2 panel A and B; *p* < 0.05].

### MIA-690 and MR-409 decrease LPS-induced cyclooxygenase (COX)-2, nuclear factor kappa-light-chain enhancer of activated B cells (NF-kB) and inducible nitric oxide synthase (iNOS) gene expression in colon specimens

**Figure 3:**
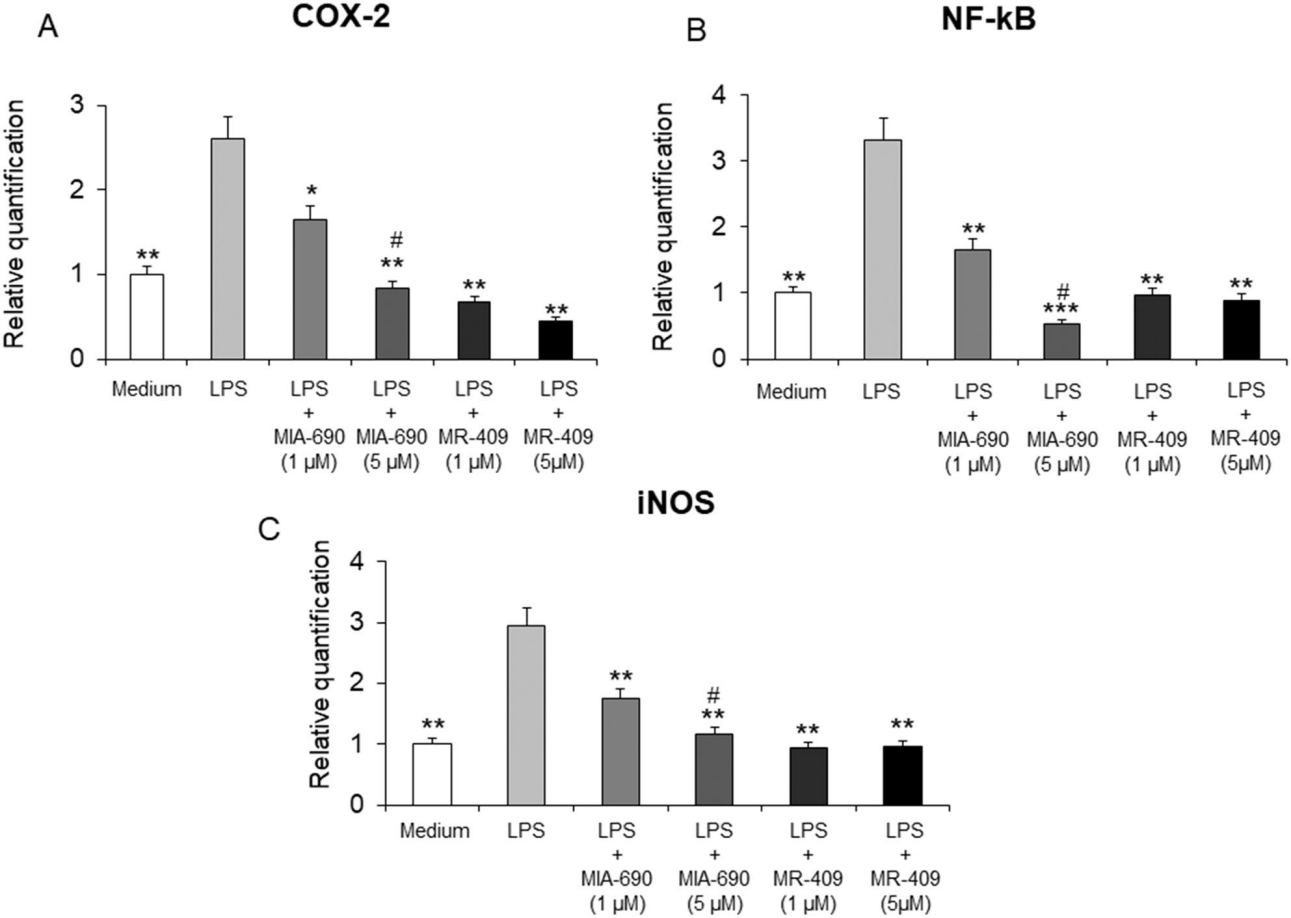
Relative quantification of COX-2 (**A**), NF-kB (**B**) and iNOS (**C**) gene expression in mouse colon specimens treated with MIA-690 (1 and 5 μM) and MR-409 (1 and 5 μM), ex vivo (n = 5 for each group of treatment). Data were calculated using the 2^-ΔΔCt^ method, normalized to β-actin mRNA levels, and expressed relative to control (calibrator sample, defined as 1.00). Data are expressed as means ± S.E.M. and analyzed by analysis of variance (ANOVA) followed by Bonferroni *post-hoc* test *p < 0.05, **p < 0.01, ***p < 0.005 versus LPS; ^#^*p* < 0.05 versus treatment with MR-409.

Real-time polymerase chain-reaction showed significantly increased gene expression of COX-2, NF-kB and iNOS in LPS-treated colon specimens, with respect to vehicle treated controls. The GHRH antagonist MIA-690 (1 and 5 μM) decreased inflammatory markers after LPS treatment in a dose-dependent manner [Fig. 3 panel A, B and C; *p* < 0.05, *p* < 0.01, and *p* < 0.005]. In addition, GHRH agonist MR-409 (1 and 5 μM) was found able to decrease LPS-induced gene expression of COX-2, NF-kB and iNOS in colon specimens, without a dose-dependent effect [Fig. 3 panel A, B and C; *p* < 0.01]. MIA-690 (5 μM) was more effective than MR-409 in inhibiting all tested markers [Fig. 3 panel A, B and C; *p* < 0.05].

### MIA-690 and MR-409 reduce nociceptive response in mice

**Figure 4:**
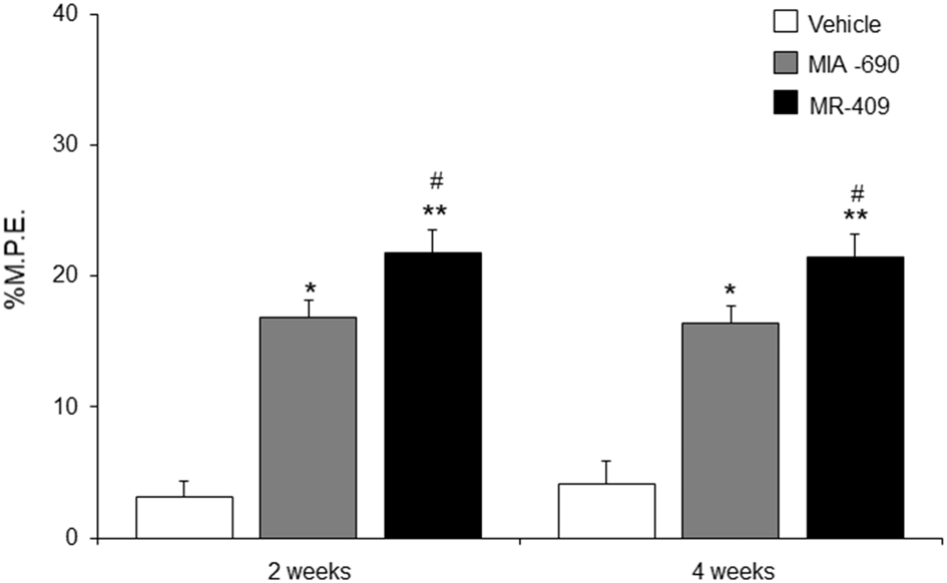
Effect of MIA-690 (5 µg) and MR-409 (5 µg) (n = 6 for each group of treatment) s.c. administration on the analgesic response at the temperature of 54.0 ± 0.4 °C in hot plate test. The antinociceptive activity is expressed as percentage of the maximum possible effect (% M.P.E.). Data are expressed as means ± S.E.M. and analyzed by analysis of variance (ANOVA) followed by Bonferroni *post-hoc* test *p < 0.05, **p < 0.01 versus vehicle; ^#^*p* < 0.05 versus MIA-690.

With the objective to determine the responsiveness to acute nociceptive stimulation, mice were tested by conventional hot plate apparatus at the temperature of 54.0 ± 0.4 °C. MIA-690 (5 μg) or MR-409 (5 μg) were s.c. treated daily for 4 weeks in mice. Control animals received s.c. injection of vehicle (0.1% DMSO (Sigma) and 10% propylene glycol). The evaluations were performed after 2 and 4 weeks of treatment. Compared to vehicle, both GHRH analogs showed maximal antinociceptive effects of 16.26% and 21.75% M.P.E. at 2 weeks [Fig. 4; *p* < 0.05 and *p* < 0.01, respectively]. Both peptides also sustained a moderate analgesic effect at 4 weeks, with maximal antinociceptive effect of 16.41 and 21.49% M.P.E. [Fig. 4; *p* < 0.05 and *p* < 0.01, respectively]. MR-409 showed greater effects respect to MIA-690 [Fig. 4; *p* < 0.05] at both 2 and 4 weeks.

### MIA-690 and MR-409 reduce responsiveness to acute inflammation stimuli

**Figure 5:**
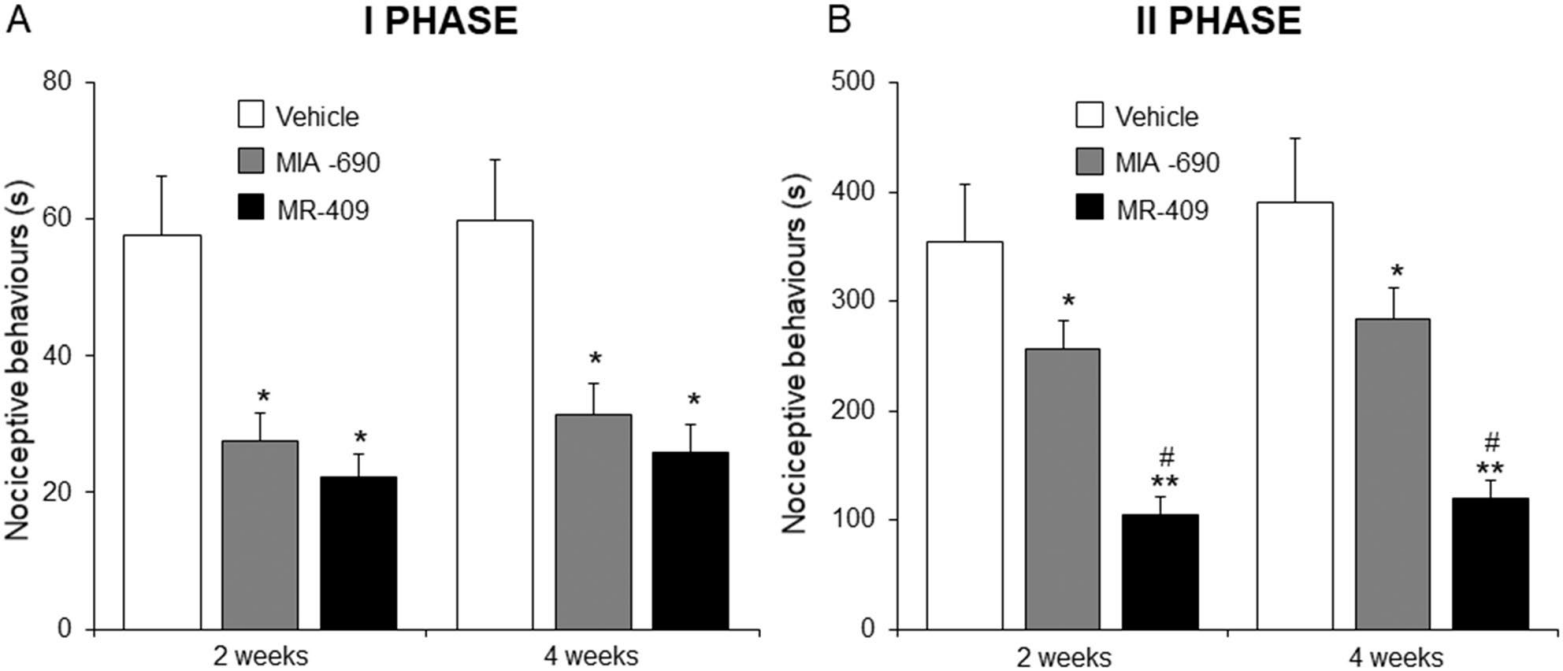
Effect of MIA-690 (5 µg) and MR-409 (5 µg) s.c. administration on the acute nociceptive response in formalin test (n = 6 for each group of treatment). The nociceptive behaviour time (seconds) of licking, shaking and biting was measured during the period of 0–5 min (first phase) and 20–60 min (second phase). Data are expressed as means ± S.E.M. and analyzed by analysis of variance (ANOVA) followed by Bonferroni *post-hoc* test *p < 0.05, **p < 0.01 versus vehicle; ^#^*p* < 0.05 versus MIA-690.

With the aim to determine the responsiveness to acute inflammation stimulation mice were tested by formalin test. MIA-690 (5 μg) or MR-409 (5 μg) were s.c. injected daily for 4 weeks in mice. Control animals received s.c. injection of vehicle (0.1% DMSO (Sigma) and 10% propylene glycol). The evaluations were performed after 2 and 4 weeks of treatment. After intraplantar injection of formalin, mice treated with MIA-690 (5 μg) or MR-409 (5 μg) showed short nociceptive behavioural responses (first and second phase) compared to vehicle groups [Fig. 4; *p* < 0.05 and *p* < 0.01], at both 2 and 4 weeks. In second phase, we showed that s.c. injection of MR-409 significantly decreased nociceptive behavioural response at both 2 and 4 weeks of treatment compared to MIA-690 [Fig. 5; *p* < 0.05].

### MIA-690 and MR-409 reduce disease activity index (DAI) score and colon length in DSS-induced colitis

**Figure 6:**
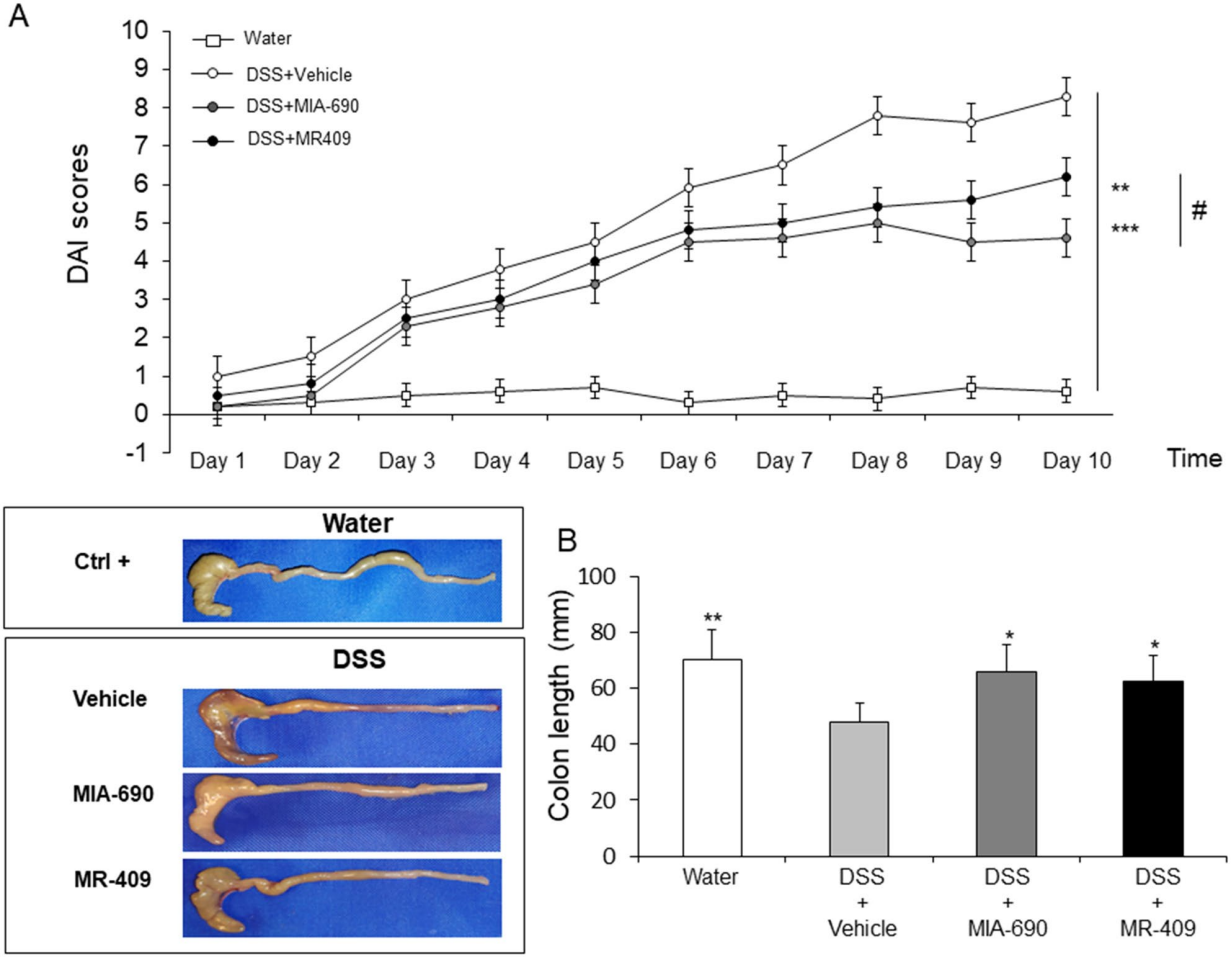
Effect of MIA-690 (5 µg) and MR-409 (5 µg) (n = 6 for each group of treatment) s.c. administration on relative DAI scores (**A**) and colon segment lengths (**B**) in a model of DSS-induced colitis. Data are expressed as means ± S.E.M. and analyzed by analysis of variance (ANOVA) followed by Bonferroni *post-hoc* test *p < 0.05, **p < 0.01, ***p < 0.005 versus DSS-treated mice.

DSS (2.5%)-induced colitis was induced to investigate the potential effects of MIA-690 (5 μg) and MR-409 (5 μg) on inflammatory stimuli. DSS treatment led to higher DAI score in all treated groups as compared to controls (mice untreated with DSS). MIA-690 (5 μg) or MR-409 (5 μg) treated mice displayed significantly lower susceptibility to DSS treatment starting from day 2 [Fig. 6, panel A; *p* < 0.01 and *p* < 0.005]. However, treatment with MIA-690 (5 μg) or MR-409 (5 μg) did not induce a significant reduction in mortality respect to vehicle treated animals (n = 1/10 for all groups; data not shown).

In our model, we observed a significant reduction in colon segment length in all groups treated with DSS respect to positive controls (mice untreated with DSS). MIA-690 (5 μg) or MR-409 (5 μg) treated mice showed a minor decrease in colon segment length respect to DSS treated animals, suggesting that both peptides induce a lower sensitivity to chronic inflammation stimuli [Fig. 6, panel B; *p* < 0.05]. No significant difference was found between MIA-690 and MR-409 treated mice in DAI score and colon segment length.

### Macroscopic and histopathological examination

**Figure 7:**
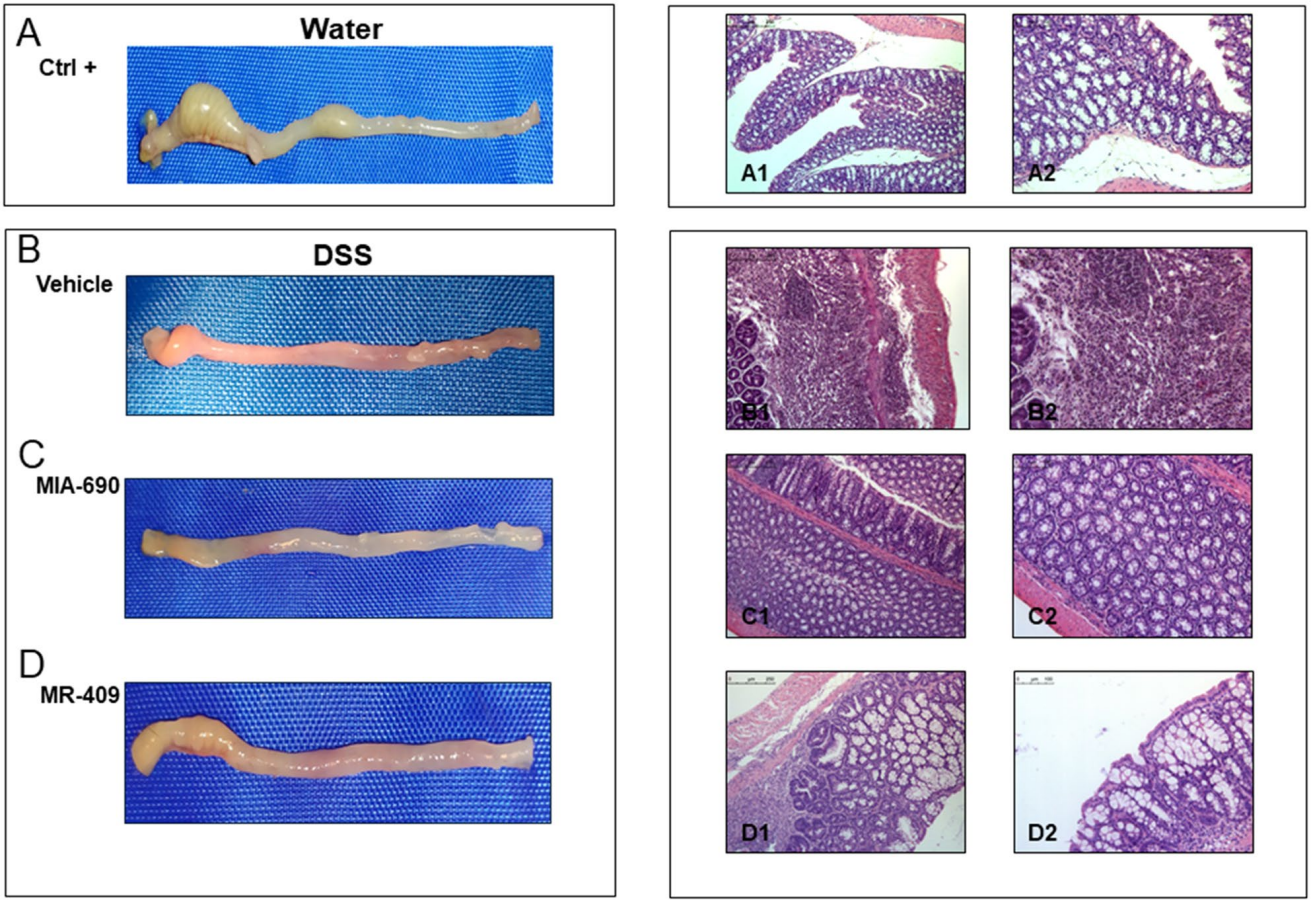
Effect of MIA-690 (5 µg) and MR-409 (5 µg) (n = 6 for each group of treatment) s.c. administration on macroscopic and histopathological changes of colon in a model of DSS-induced colitis. A (a1 and a2): normal colon segment; B (b1 and b2), C (c1 and c2) and D (d1 and d2): inflammation of colon segment in DSS-induced colitis; necrosis of epithelium, distortion of crypts, inflammation, infiltrates in lamina propria and submucosa as well as submucosal edema. Hematoxilyn and eosin stain: the inserted bars indicate magnification. [H&E staining, × 10 (a1, b1, c1, d1); × 20 (a2, b2, c2, d2) original magnification].

Representative macroscopic and histological images of H&E-stained colon sections from each group are shown in Fig. 7. Respect to normal colon (positive control Ctrl; Fig. 7A, panel a1 and a2), we observed high (vehicle; Fig. 7B, panel b1 and b2), low (MIA-690; Fig. 7C, panel c1 and c2) and moderate (MR-409; Fig. 7D, panel d1 and d2) inflammation. In positive control (Ctrl; Fig. 7B, panels b1 and b2 and) we found that DSS treated mice showed an important necrosis of the epithelium and submucosal edema in positive control. These changes were associated with marked infiltration of inflammatory cells in the lamina propria and submucosa. The infiltrated inflammatory cells included polymorphonuclear leucocytes, lymphocytes, and plasma cells.

In addition, histological analysis showed lower inflammation in mice treated with MIA-690 (5 µg) (Fig. 7 C, panel c1 and c2). Despite a weak infiltration of lymphocytes into the mucosal tissue, crypt damage was not observed. Moreover, the animals treated with MR-409 (5 µg) showed moderate lymphocytic infiltrate and important crypt destruction in histological examination (Fig. 7D; panel d1 and d2).

### MIA-690 and MR-409 decreased PGE_2_ and 8-iso-PGF_2α_ levels in colon specimens of DSS-induced colitis

**Figure 8:**
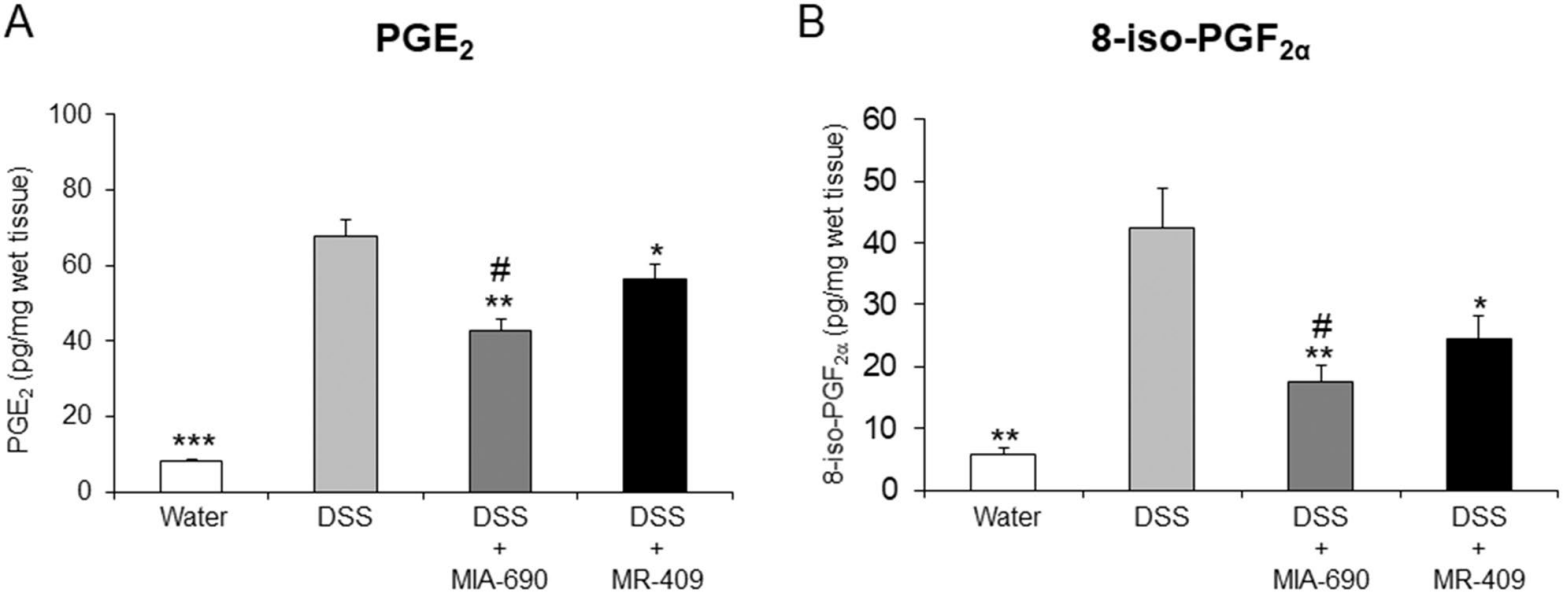
Inhibitory effects of MIA-690 (5 g) and MR-409 (5 μg) on prostaglandin E_2_ (**A**) and 8-iso-prostaglandin F_2α_ (**B**) levels in colon segment of DSS-induced colitis (n = 6 for each group of treatment). Water group were control mice untreated with DSS. Data are expressed as means ± S.E.M. and analyzed by analysis of variance (ANOVA) followed by Bonferroni *post-hoc* test *p < 0.05 **p < 0.01,***p < 0.005 versus water controls; ^#^*p* < 0.05 versus MR-409 treated mice.

In order to study the possible effects of MIA-690 (5 μg) and MR-409 (5 μg) on inflammatory and oxidative stress biomarkers, we determined PGE_2_ and 8-iso-PGF_2α_ levels in colon specimens from DSS-treated mice. DSS treatment significantly stimulated both PGE_2_ and 8-iso-PGF_2α_ levels, and both MIA-690 (5 µg) and MR-409 (5 µg) reduced these effects, with MIA-690 more active respect to MR-409 [Fig. 8; *p* < 0.005; *p* < 0.01 and *p* < 0.05].

### Effects of MIA-690 and MR-409 on serotonin (5-hyroxytriptamine, 5-HT) and kynurenic acid (KA) levels in colon specimens of DSS-induced colitis mice

**Figure 9:**
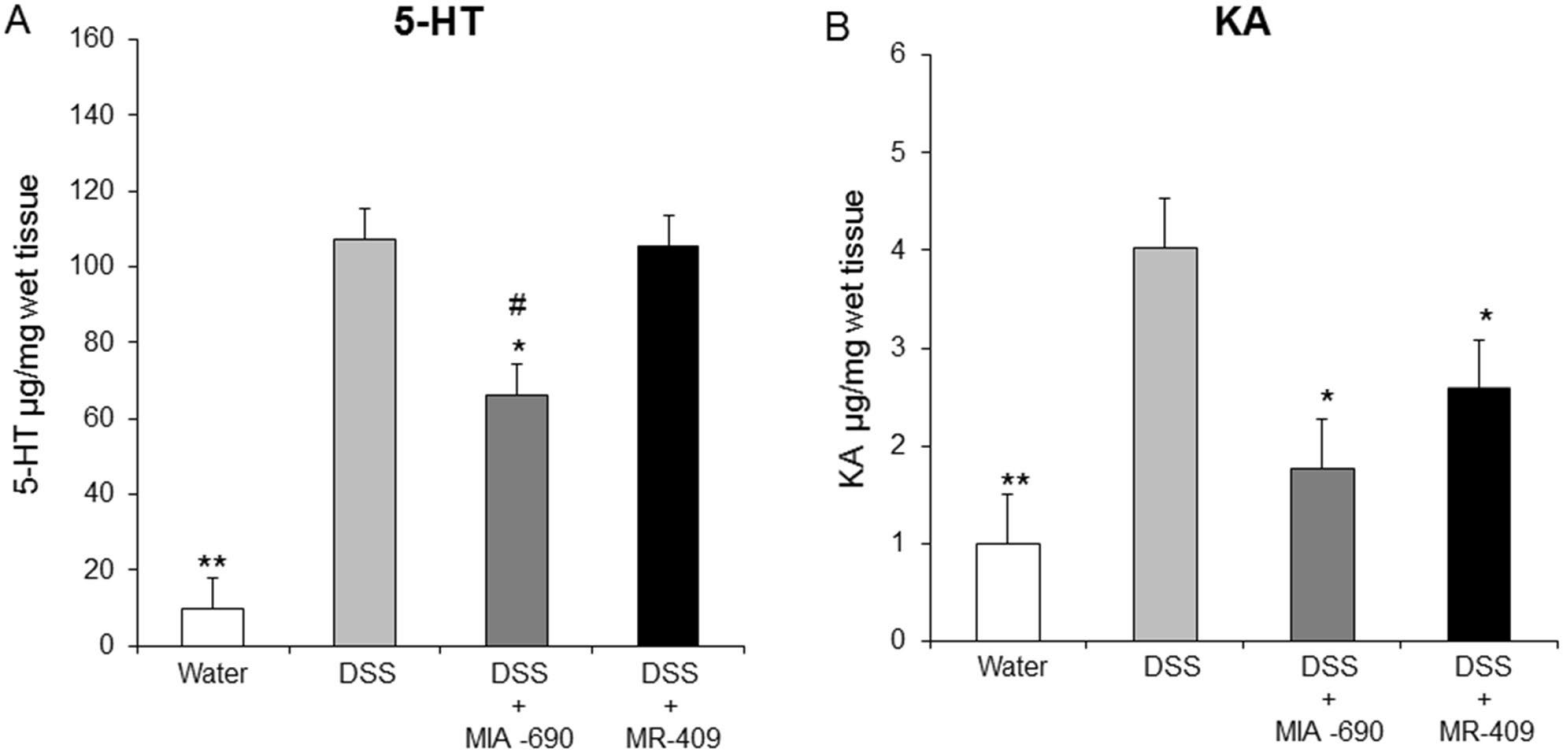
Effects of MIA-690 (5 µg) and MR-409 (5 μg) on 5-HT (**A**) and KA (**B**) levels in colon segments of DSS-induced colitis mice (n = 6 for each group of treatment). Data are expressed as means ± S.E.M. and analyzed by analysis of variance (ANOVA) followed by Bonferroni *post-hoc* test *p < 0.05 **p < 0.01 versus DSS treated mice; ^#^*p* < 0.05 versus MR-409 treated mice.

We also investigated the potential effects of MIA-690 (5 μg) and MR-409 (5 μg) on 5-HT and KA production in colon specimens from DSS-treated mice. In this context, s.c. administration of MIA-690, but not MR-409, significantly decreased 5-HT levels [Fig. 9; *p* < 0.05]. On the other hand, both peptides significantly reduced KA levels [Fig. 9; *p* < 0.05].

### Effects of MIA-690 and MR-409 on tumor necrosis factor (TNF)-α, interleukin (IL)-6 and iNOS gene expression in colon specimens of DSS-induced colitis mice

**Figure 10:**
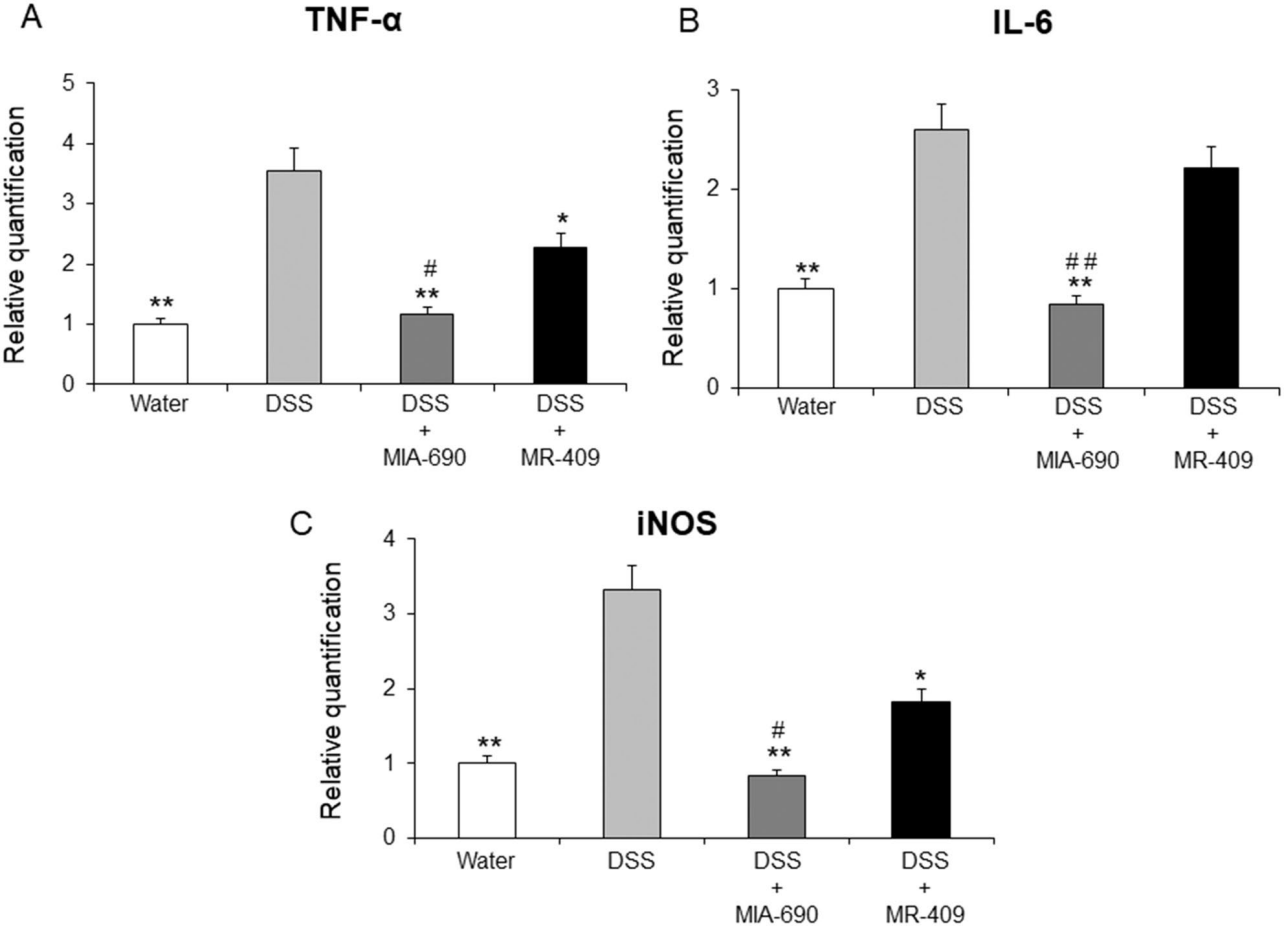
Effects of MIA-690 (5 μg) and MR-409 (5 μg) on relative gene expression of TNF-α, IL-6 and iNOS in colon segment of DSS-treated mice (n = 6 for each group of treatment) as determined by real-time RT PCR. Data were calculated using the 2^−ΔΔCt^ method; normalized to β-actin mRNA levels and then expressed as relative to control (calibrator sample, defined as 1.00). Data are expressed as means ± S.E.M. and analyzed by analysis of variance (ANOVA) followed by Bonferroni *post-hoc* test *p < 0.05 and **p < 0.01 versus DSS treated mice; ^#^*p* < 0.05 and ^##^*p* < 0.01 versus MR-409 treated mice.

Real-time polymerase chain-reaction showed a significant increase in TNF-α, IL-6 and iNOS mRNA levels in all groups, as compared with controls. MIA-690 (5 µg) decreased TNF-α, IL-6 and iNOS gene expression, while MR-409 (5 µg) did not modify IL-6 and was less effective than MIA in reducing TNF-α, and iNOS [Fig. 10; *p* < 0.05, *p* < 0.01].

### Effects of MIA-690 and MR-409 on serum insulin-like growth factor (IGF)-1 levels

**Figure 11:**
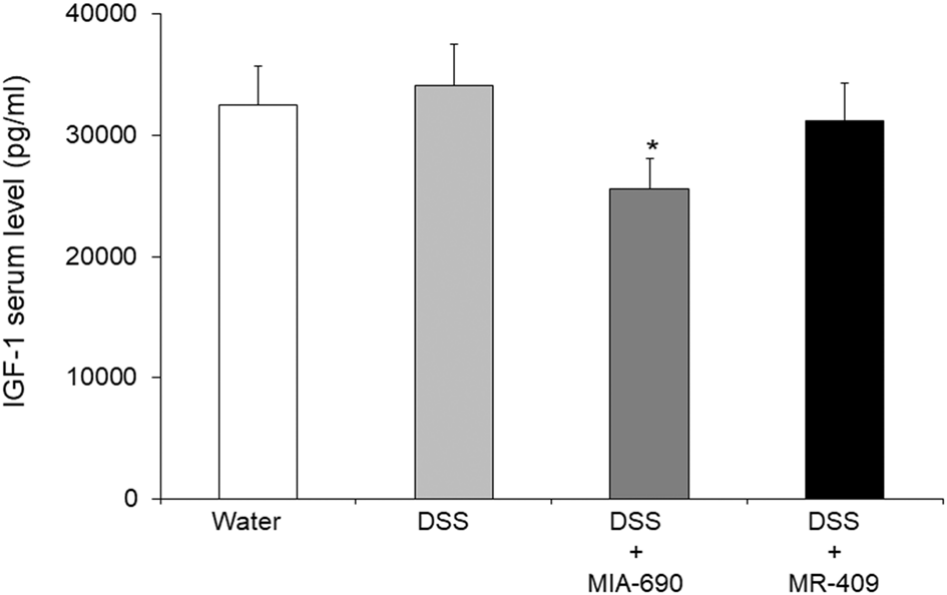
Effects of MIA-690 (5 μg) and MR-409 (1-5 μM) on IGF-1 levels in serum of DSS-treated mice (n = 6 for each group of treatment) as determined by ELISA kit. Data are expressed as means ± S.E.M. and analyzed by analysis of variance (ANOVA) followed by Bonferroni *post-hoc* test *p < 0.05 versus water controls, DSS or DSS + MR-409 treated mice.

ELISA results showed a significant reduction in circulating levels of IGF-1 in MIA-690 (5 μg) treated mice, but not in MR-409 (5 μg) treated mice (Fig. 11; *p* < 0.05).

## Discussion

Our present findings showed that MIA-690, a GHRH antagonist, and MR-409, an agonistic GHRH analog, exert antinflammatory and antioxidant effects on colon specimens, ex vivo (Figs. 1, 2 and 3). In agreement, a number of studies demonstrated that GHRH and GHRH antagonists can modulate the inflammatory and reduction/oxidation (redox) status in cancer and other tissues^22,23^. In particular, MIA-690 has proved able to decrease inflammation by suppressing the infiltration of macrophages and leucocytes, and the secretion of TNF-α, IL-1β, and monocyte chemotactic protein-1 (MCP-1) in aqueous humor after insult with LPS^24^. Moreover, MIA-690 exerted antinflammatory and antioxidant effects in different experimental paradigms^25,26^. MIA-690 was also found able to decrease the expression of proinflammatory and oxidative markers, such as COX-2, TNF-α, NF-kB, iNOS and Nrf2, in ex vivo and in vivo studies^26^. Similarly, MR-409 also showed antinflammatory and antioxidant effects^26^, with neural and vascular protective properties in early experimental diabetic retinopathy^27^. Interestingly, the authors hypothesized that the beneficial effects induced by MR-409 could be direct and/or GH-mediated.

Firstly, the responsiveness to nociceptive and acute inflammation stimuli was evaluated following chronic administration of MIA-690 and MR-409 in vivo. We found that both peptides induce analgesic and antinflammatory effects after chronic treatment, both at 2 and 4 weeks of treatment (Figs. 4, 5). The role of the somatotropic axis in analgesia is contradictory, with scant data relative to GHRH. Receptors for GH and IGF-1 and -2 are expressed in the hippocampus, a brain region involved in pain perception^28^ and various studies have reported the link between endogenous opiates and GHRH in the human hypothalamus^29^. Interestingly, both GHRH agonist and antagonist analogs showed similar antinociceptive activities. However, the agonist peptide MR-409 showed higher antinociceptive effects with respect to the antagonist MIA-690 at both 2 and 4 weeks (Fig. 4). GHRH has been shown to bind to opioid receptors^30^, and we have previously shown that generalized ablation of the GHRH gene (GHRHKO) increases sensitivity to thermal pain, in mice^31^.

On the other hand, mice treated with both peptides showed also a lower responsiveness after intraplantar formalin injection (Fig. 5). The involvement of the somatotropic axis in inflammatory states is well known^32,33^. Various agonist and antagonist analogs of GHRH have shown marked antinflammatory effects^16,19,27,34–36^. In particular, MIA-690 inhibited inflammation by reducing the production of inflammatory markers in carrageenan-induced chronic prostatitis^35^. Moreover, pre-treatment with GHRH was found to suppress endotoxin-induced inflammatory hyperalgesia in animal models^37^. In addition, GHRHKO mice showed a significantly increased sensitivity to inflammatory stimuli^31^.

These data have allowed us to perform our study on DSS-induced colitis.

Finally, in our model of DSS-induced colitis, animals treated with both MIA-690 and MR-409 displayed lower DAI score and a significant increase in colon segment length as compared with vehicle (Fig. 6). However, we did not observe any difference in mortality between groups, probably due to the short duration of treatment with DSS. In this context, the antinflammatory effects of MIA-690 were more marked respect to MR-409. MIA-690 decreased the infiltration of lymphocytes into mucosal tissue (Fig. 7, panel c1 and c2), PGE_2_, 8-iso-PGF_2α_ and 5-HT levels (Figs. 8 and 9), as well as TNF-α, IL-6 and iNOS gene expression (Fig. 10) in colon specimens. Despite both peptides showed similar effects on DAI score and colon segment length, MR-409 did not significantly decrease the inflammatory response (Fig. 7). In particular, histological analysis showed a moderate increase of crypt destruction and infiltration of lymphocytes in the mucosa, with respect to vehicle or MIA-690 treatment (Fig. 7, panel d1 and d2). However, MR-409 decreased all inflammatory markers, except 5-HT and IL-6 (Figs. 9 and 10). Our results are in agreement with those of Ren and collaborators (2019) showing that another GHRH antagonist of MIAMI series, MIA-602, was found able to suppress elevated expression of some pro-inflammatory cytokines, including IL-6, and iNOS, in ciliary and iris epithelial cells during LPS-induced ocular inflammation; while these effects were not observed for MR-409^38^.

5-HT is involved in the pathogenesis of intestinal disorders, including IBD^39–41^, irritable bowel syndrome^39,42,43^ and DSS-induced experimental colitis^44^. In addition, IL-6 is up regulated in colitis^45–48^ and it has been shown to be positively correlated with disease severity in IBD^49^. The lower antinflammatory effects of MR-409 with respect to MIA-690 could be explained by lack of effects on 5-HT and IL-6 levels.

IGF-1, in addition to its well-established growth and metabolic effects, is also involved in inflammatory processes and immune cell recruitment^50^. In particular, IGF‐1 enhances intestinal inflammation and inflammation‐associated tumorigenesis^50^. Following MIA-690 treatment in mice, we found a reduction of serum IGF-1 levels (Fig. 11). In this context, Schally and collaborators (2008) reported that GHRH antagonists suppressed the release of GH from pituitary through binding to the GHRH receptors and blocked the production and release of GH^16^. Thereafter, the reduction in serum IGF-1 could be a consequence of blocking the secretion of GH, as previously observed in other works^20,25^. The IGF‐1 pathway has been finely related to cancer and IBD, by regulating the immune system and through its multifunctional involvement in tumor microenvironment^51^. Aberrant IGF‐1 signaling has been observed in several cancers in humans, with the involvement of IGF‐1 receptor signaling in cancer cell proliferation, migration, and invasion as well as in resistance to therapeutic agents^50,52–55^.

On the other hand, MR-409 did not affect serum IGF-1 levels. Our findings could be explained by a transient stimulatory effect induced by MR-409 on GH release, previously reported by Cui and Schally (2018)^56^. A reduction in serum IGF-1 levels was shown from 24 h on day 1 to day 5, in C57BL/6 mice treated with MR-409 (5 μg/day) for 15 days^56^. However, after day 10, no change was found in serum levels of IGF-1 in the animals treated with MR-409 compared to controls^56^. Accordingly, except for the first hour, no significant difference in GH levels was shown between MR-409- and control-treated mice^56^. In addition, no significant change in serum GH levels was previously reported in MR-409-treated animals bearing three types of lung cancer^19^. Our results are also in agreement with those of Qin and collaborators (2014) showing that IGF-1 levels in aqueous humor was significantly decreased after treatment with MIA-690, but not MR-409, in experimental ocular inflammation^57^. Moreover, GHRH agonists did not modify serum levels of IGF-1 in rats following 4 week-treatment, compared to placebo-treated animals^58,59^. On the other hand, GH/IGF-1 independent effects were previously reported for GHRH agonists, including MR-409^58,59^. The inhibitory effects of MIA-690 on serum IGF-1 levels could partially explain the higher antinflammatory and antioxidant effects displayed by this peptide as compared to MR-409.

In conclusion, both GHRH antagonist MIA-690 and agonist MR-409 show antinflammatory and antioxidant properties in ex vivo experimental models. Both peptides also decrease thermal sensitivity and chemical responsiveness to acute and persistent inflammatory stimuli, in in vivo studies. The higher antinflammatory and antioxidant activities observed for MIA-690 could be related to decreased serum IGF-1 levels. However, we can not rule out a possible involvement of GH/IGF-1 independent effects in modulation of protective activities induced by MIA-690.

## Methods

### Peptides and chemicals

The GHRH antagonist MIA-690 and agonist MR-409 were synthesized by R.C. and W.S. in the laboratory of one of us (A.V.S.). For ex vivo studies, the peptides were dissolved in DMSO to form a 5 mM solution, and then further diluted to the concentration indicated. For in vivo studies, the peptides were dissolved in an aqueous solution of 0.1% DMSO (Sigma) and 10% propylene glycol (Sigma-Aldrich, St. Louis, MO)^19,25^.

### Animals

Adult C57/BL6 male mice (5 weeks old, weight 20–22 g, n = 48 were housed in Plexiglas cages (2–4 animals per cage; 55 × 33 × 19 cm), on a 14/10 h light/dark cycle, with ad libitum access to water and food. Only male mice were used to avoid any possible involvement of hormonal changes in adult female mice. Mice were fed with a standard rodent chow (Prolab RMH2500, PMI Nutrition International, Brentwood, MO). Housing conditions and experimentation procedures were strictly in agreement with the European Community ethical regulations (EU Directive n. 26/2014) on the care of animals for scientific research. The study was carried out in compliance with the ARRIVE guidelines. In agreement with the recognized principles of “Replacement, Refinement and Reduction of Animals in Research”, colon specimens were obtained as residual material from vehicle-treated mice randomized in our previous experiments approved by Local Ethical Committee (‘G. d’Annunzio’ University, Chieti-Pescara) and Italian Health Ministry (Project n. 885/2018-PR).

### Ex vivo studies

Mice were sacrificed by CO_2_ inhalation (100% CO_2_ at a flow rate of 20% of the chamber volume per min) and colon was immediately collected and maintained in a humidified incubator with 5% CO_2_ at 37 °C for 4 h (incubation period), in RPMI buffer with added bacterial LPS (10 μg/mL), as previously described^60^. During the incubation period, the tissues were treated with MR-409 or MIA-690 (1–5 μM). Tissue supernatants were collected and prostaglandin (PG) E_2_ and 8-iso-PGF_2α_ levels (pg/mg wet tissue) were measured by radioimmunoassay (RIA), as previously reported^60^. Briefly, specific anti-PGE_2_ and anti-8-iso-PGF_2α_ were developed in the rabbit; the cross-reactivity against other prostanoids was < 0.3%. One hundred microliters of prostaglandin standard or sample was incubated overnight at 4 °C with the^3^H-prostaglandin (3000 cpm/tube; NEN) and antibody (final dilution: 1:120 000; kindly provided by the late prof. G. Ciabattoni), in a volume of 1.5 mL of 0.025 M phosphate buffer. Free and antibody-bound prostaglandins were separated by the addition of 100 μL 5% bovine serum albumin and 100 μL 3% charcoal suspension, followed by centrifugation for 10 min at 4000 g at 5 °C and decanting off the supernatants into scintillation fluid (UltimaGold Perkin Elmer) for β emission counting. The detection limit of the assay method was 0.6 pg/mL. Tissue supernatants were also assayed for nitrite determination by Griess assay, as previously described^26^. Briefly, nitrite production was determined by mixing 50 μL of the assay buffer with 50 μL of Griess reagent (1.5% sulfanilamide in 1 M HCl plus 0.15% N-(1-naphthyl) ethylenediamine dihydrochloride in distilled water, v/v). After incubation for 10 min, at room temperature, the absorbance at 540 nm was determined and nitrite concentrations were calculated from a standard curve for sodium nitrite.

Tissue supernatants were also assayed for LDH activity^26^. LDH activity was measured by evaluating the consumption of NADH in 20 mM HEPES-K + (pH 7.2), 0.05% bovine serum albumin, 20 μM NADH and 2 mM pyruvate using a microplate reader (excitation 340 nm, emission 460 nm) according to manufacturer's protocol (Sigma-Aldrich, St. Louis, MO). LDH activity was measured by evaluating the consumption of NADH in 20 mM HEPES-K + (pH 7.2), 0.05% bovine serum albumin, 20 μM NADH and 2 mM pyruvate using a microplate reader (excitation 340 nm, emission 460 nm) according to manufacturer's protocol. Nitrite and LDH production data were expressed as relative variations compared to vehicle-treated specimens. Immediately after sacrifice, colon specimens were rapidly removed, dissected and stored in RNAlater solution (Ambion, Austin, TX) at -20 °C until further processed. Total RNA was extracted from the colon specimens using TRI Reagent (Sigma-Aldrich), according to the manufacturer’s protocol. One microgram of total RNA extracted from each sample in a 20 µl reaction volume was reverse transcribed using High Capacity cDNA Reverse Transcription Kit (Applied Biosystems). The samples were incubated in a 2720 Thermal Cycler (Applied Biosystems) initially at 25 °C for 10 min, then at 37 °C for 120 min, and finally at 85 °C for 5 s. Gene expression of COX-2, NF-kB and iNOS was determined by quantitative real-time PCR using TaqMan probe-based chemistry (Applied Biosystems), as previously described^61,62^. PCR primers and TaqMan probes were obtained from Applied Biosystems [Assays-on-Demand Gene Expression Products, Mm00478374_m1 for COX-2 gene, Mm00476361_m1 for NF-kB gene, Mm00440502_m1 for iNOS gene, Mm00607939_s1 for β-actin gene]. β-actin was used as the housekeeping gene. Gene expression data were calculated as previously reported^63^.

### In vivo studies

After 2-week acclimation, mice were treated daily by subcutaneous (s.c.) administration of 0.1 ml solution of GHRH antagonist MIA 690 (5 µg), GHRH agonist MR 409 (5 µg) or vehicle^20^. All solutions were freshly prepared before use. The concentrations were selected based on previous studies^19,20^. All treatments were administered at 09:00 a.m., and all in vivo testings were performed between 10:00 a.m. and 12:00 a.m. to minimize circadian variations in sensitivity to pain^64^. Care was taken to standardize testing conditions^65^. The experimental room was maintained with minimal background noise and constant temperature (22 ± 2 °C). All materials used for each test were thoroughly cleaned after test completion for each mouse. At the end of each test, the animals were returned to their home cages, and the apparatus was cleaned with 75% ethanol and dried before the next procedure. Antinociceptive and antinflammation testing was performed at 2 and 4 weeks after the first injection. Each test was conducted on the same group of animals (n = 6 for each group of treatment), after a 2-week rest period to avoid any interference on behavioral test performance, as previously reported^66^.

### Hot plate test

Basal responsiveness to nociceptive stimulation was measured by hot-plate apparatus (2biological Instruments, Varese, Italy), set at the temperature of 54.0 ± 0.4 °C, as previously described^31^. The antinociceptive response was the latency from placement of the mouse on the heated surface until the first overt behavioral sign of nociception, such as (i) licking a hind paw, (ii) vocalization, or (iii) an escape attempt (jumping off the plate). The timer was stopped by a foot-operated pedal and the mouse was immediately removed from the hot-plate immediately after responding or after a maximum of 30 s (cut-off), to prevent tissue damage. In our conditions, the baseline latencies obtained in pre-experimental tests, ranged from 5.1 ± 0.7 to 6.2 ± 0.8 s. The analgesic effect was calculated as a percentage of the maximum possible effect (%M.P.E.) according to the formula: (TL-BL)/(30-BL) × 100, where TL = test latency, BL = baseline latency, 30 = cut-off time in seconds.

### Formalin test

A diluted formalin solution was injected subcutaneously under the plantar surface of a hind paw, and pain related behaviors were scored during two successive phases^31^. The first phase (0–5 min.) reflects direct activation of nociceptors and therefore provides a measure of acute chemical pain. The second phase (20–60 min) mainly reflects persistent pain that is associated with developing inflammatory response within the injected paw. Each mouse was placed in a transparent Plexiglas box (17.5 × 23.5 × 9.5 cm) positioned above a mirror to allow an unobstructed view of the paw. After a 15-min habituation period, formalin (10 μl of a 3% solution in saline) was injected under the plantar surface of the right hind paw, using a 50 μl Hamilton microsyringe with a 25-gauge needle. The mouse was placed back into the box immediately after the injection and behavioral scoring consisted of either counting the number of nociceptive responses (paw licking, shaking and biting) or measuring how long the animal produced these responses.

### DSS-induced colitis

Male mice were randomized into three groups and treated daily for 7 days. Colitis was induced by 2.5% (w/v) DSS (molecular weight 40 kDa; Sigma Aldrich, Steinheim, Germany) added to the drinking water, ad libitum for 7 days^31^. C57BL/6 (n = 6) mice untreated with DSS were used as positive control.

### Colitis DAI analysis

Colitis DAI scoring was calculated as previously described^67^. DAI score was the combined score of weight loss (0, none; 1, 0–5%; 2, 5–10%; 3, 10–20%; and 4, > 20%), stool consistency change (0, none; 2, loose stool; and 4, diarrhea), and bleeding (0, none; 1, trace; 2, mild hemoccult; 3, obvious hemoccult; and 4, gross bleeding), and then divided by three. The minimal score was 0 and the maximal score was 4. The animals were scored for the DAI at the same time of each day, and DAI score was recorded every day. Animals were treated with anesthetic and analgesic drugs [caprofen 10 mg/kg; meloxicam 10 mg/kg; lidocaine (1–2%) 2–4 mg/kg] when they displayed signs of distress, according to the guidelines suggested by the ‘National Centre for the Replacement, Refinement and Reduction of Animals in Research’ (NC3RS).

### Macroscopic and histological evaluation

Mice were sacrificed by CO_2_ inhalation (100% CO_2_) at a flow rate of 20% of the chamber volume per min), individual colon was dissected and the colon segment length (from ileocecal junction to the anal verge; mm) measured by a ruler. Tissues were fixed in 10% phosphate-buffered formalin, dehydrated in a series of alcohol solutions of 50%, 70%, 95% and 99% and then in xylene. Samples were then paraffin-embedded and cut in 5 μm-thick sections. Sections were de-waxed (xylene and alcohol in progressively lower concentrations), rehydrated and processed for Hematoxylin and Eosin (H&E) staining (Bio Optica, Milano, Italy) according to the manufacturer protocol.

H&E-stained 5 mm distal colonic sections were coded for blind microscopic assessment of inflammation. Samples were then observed by means of Leica DM 4000 microscope (Leica Cambridge Ltd, Cambridge, UK) equipped with a Leica DFC 320 camera (Leica Cambridge Ltd.) for computerized images.

### PGE_2_ and 8-iso-PGF_2α_ production in isolated colon specimens

PGE_2_ and 8-iso-PGF_2α_ levels (ng/mg wet tissue) in isolated colon specimens of mice treated with MIA-690 (5 µg) andMR-409 (5 µg) were evaluated by radioimmunoassay (RIA), as previously reported^31^. Colon specimens dissected from C57BL/6 (n = 6) mice untreated with DSS were used as positive control.

### 5-HT and high performance liquid chromatography (HPLC) determination

Tissue 5-HT levels were analyzed through an HPLC apparatus consisting of a Jasco (Tokyo, Japan) PU-2080 chromatographic pump and an ESA (Chelmsford, MA, USA) Coulochem III coulometric detector, equipped with microdialysis cell (ESA-5014b) porous graphite working electrode and solid-state palladium reference electrode. The analytical conditions for identification and quantification were selected according to a previous study. Briefly, the analytical cell was set at -0.150 V, for detector 1 and at + 0.300 V for detector 2, with a range of 100 nA. The chromatograms were monitored at the analytical detector 2. Integration was performed by Jasco Borwin Chromatography software, version 1.5. The chromatographic separation was performed by isocratic elution on Phenomenex Kinetex reverse phase column (C18, 150 mm × 4.6 mm i.d., 2.6 µm). As regards separation of 5-HT, the mobile phase was (10:90 v/v) acetonitrile and 75 mM pH 3.00 phosphate buffer containing octanesulfonic acid 1.8 mM, EDTA 30 µm and triethylamine 0.015% v/v. Flow rate was 0.6 ml/min and the samples were manually injected through a 20 µl loop. Analyte peaks were identified by comparison with the retention time of pure standard. Analyte concentrations in the samples were calculated by linear regression curve (y = bx + m) obtained with standard. The standard stock solution of 5-HT at 2 mg/ml was prepared in bidistilled water containing 0.004% EDTA and 0.001% sodium bisulfite. The stock solutions were stored at 4 °C. Work solutions (1.25–20.00 ng/ml) were obtained by progressively diluting the stock solutions in the mobile phase.

### KA and HPLC-Fluorimetric Determination

The KA quantitative determination in the tissue was carried out a reversed phase HPLC-fluorimeter in agreement with the method employed by Pocivavsek and colleagues. Analyses were performed by using a liquid chromatograph (MOD. 1525, Waters Corporation, Milford MA, USA) equipped with a fluorimetric detector (MOD, 2475, Waters Corporation), a C18 reversed-phase column (Acclaim 120,3 µm, 2.1 × 100 mm, Dionex Corporation Sunnyvale, CA, USA), and an on-line degasser (Biotech 4-CH degasi compact, LabService, Anzola, Italy). The separation was conducted in isocratic conditions and the mobile phase consisted of 250 mM zinc acetate, 50 mM sodium acetate, and 3% aceto nitrile (pH adjusted to 6.2 with glacial acetic acid), using a flow rate of 1.0 mL/min. In the eluate, the KA was identified and measured fluorimetrically (excitation: 344 nm; emission: 120 nm).

### RNA extraction, reverse transcription and real-time reverse transcription polymerase chain reaction (real-time RT PCR)

Colon tissue was rapidly removed, dissected and stored in RNAlater solution (Ambion, Austin, TX) at -20 °C until further processed as previously described. Gene expression of TNF-α, IL-6 and iNOS was determined by quantitative real-time PCR using TaqMan probe-based chemistry (Applied Biosystems, Foster City, CA, USA). PCR primers and TaqMan probes were obtained from Applied Biosystems (Assays-on-Demand Gene Expression Products, Mm00443258_m1 for TNF-α gene, Mm00446190_m1 for IL-6 gene, Mm00440502_m1 for iNOS gene, Mm00607939_s1 for β-actin gene. β-actin was used as the housekeeping gene. Gene expression data were calculated as previously reported^63^.

### IGF-1 Analysis

Blood sample (1 ml) collection was performed through cardiac puncture in mice. Serum, obtained by centrifugation of total blood at 450 g at 4 °C for 15 min, was stored at − 80 °C until analysis. IGF-I were measured using mouse IGF-I ELISA Kit (Abcam, cod: ab100695), following the manufacturers’ instructions. Results were assessed by colorimetric detection at 450 nm absorbance using LT-4000 microplate reader (Euroclone).

### Statistical analysis

Statistical analysis was performed using GraphPad Prism version 5.01 for Windows (GraphPad Software, San Diego, CA, USA). All data were collected from each of the animals used in the experimental procedure and means ± SEM were determined for each experimental group and analyzed by two-way analysis of variance (ANOVA) followed by Bonferroni post-hoc test. F values are referring to repeated measure 2-way ANOVA. As for gene expression analysis, 1.00 (calibrator sample) was considered the theoretical mean for the comparison. Statistical significance was accepted at *p* < 0.05. As regards gene expression analysis, the comparative 2^-ΔΔCt^ method was used to quantify the relative abundance of mRNA and then to determine the relative changes in individual gene expression (relative quantification)^63^. Number of animals randomized for each experimental group was calculated on the basis of the ‘Resource Equation’ N = (E + T)/T (10 ≤ E ≤ 20)^68^, according to the guidelines suggested by the ‘National Centre for the Replacement, Refinement and Reduction of Animals in Research’ (NC3RS) and reported on the following web site: https://www.nc3rs.org.uk/experimental-designstatistics.
